# Screening for potential familial hypercholesterolaemia in general practice: an observational study on prevalence and management

**DOI:** 10.3399/bjgpopen20X101142

**Published:** 2021-02-03

**Authors:** Stefan Mülverstedt, Per Rossen Hildebrandt, Eva Prescott, Merete Heitmann

**Affiliations:** 1 Department of Cardiology, Copenhagen University Hospital, Bispebjerg, Copenhagen, Denmark; 2 Heart Clinic Frederiksberg, Frederiksberg, Denmark

**Keywords:** familial hypercholesterolaemia, mass screening, general practice, lipid lowering therapy, cardiovascular diseases

## Abstract

**Background:**

Familial hypercholesterolaemia (FH) is a common genetic disorder causing premature cardiovascular disease (CVD). The estimated prevalence of probable or definite FH is 1:200–250 individuals, according to the Dutch Lipid Clinic Network (DLCN) criteria for FH. In Denmark approximately 12% of cases are identified.

**Aim:**

To provide knowledge of the prevalence and management of FH in general practice.

**Design & setting:**

A collaboration between six general practice clinics and the department of cardiology at Bispebjerg hospital in Denmark.

**Method:**

A total of 9652 patient records were screened for hypercholesterolaemia. All patients with a low-density lipoprotein cholesterol (LDL-C) ≥5.0 mmol/l were included in the study population and their records were investigated in order to perform a diagnostic score according to the DLCN criteria.

**Results:**

It was found that 2382 individuals had a lipid measurement available, and 236 of those had an LDL-C ≥5.0 mmol/l. In total, 34 individuals were found to have probable or definite FH (DLCN score ≥5). Only three individuals had been diagnosed and treated with lipid-lowering therapy. Of 236 individuals with high LDL-C, only 25 individuals met their treatment target. By excluding patients with signs of secondary hypercholesterolaemia, a subgroup of 115 individuals with potential primary hypercholesterolaemia was established. Among those, 21 individuals were found to have probable or definite FH (1:114 individuals).

**Conclusion:**

The study shows that there is a massive lack of recognition of FH in general practice. Despite a measured high LDL-C, the diagnosis is rarely made and only a few patients are treated accordingly. Of the patients undergoing treatment, only a few reached their treatment target.

## How this fits in

FH is significantly underdiagnosed in general practice. This study therefore aimed to establish the extent of FH diagnosis in general practice in Denmark. Referral of potential patients with FH was not sufficient, even when FH was suspected by the GP. Individuals suspected of FH did not meet their LDL-C treatment target. Only half of the population with LDL-C ≥5 mmol/l underwent lipid-lowering therapy.

## 



## Introduction

FH is a common genetic disorder that causes premature coronary heart disease and myocardial infarction (MI), which is because of a lifelong elevated level of LDL-C. If left untreated, individuals with heterozygous FH often develop coronary artery disease at an early age.^1,2^

The lack of recognition and undertreatment of individuals with FH in the general population is largely unknown worldwide, but based on assessment of 98 098 individuals in the Copenhagen General Population Study, FH-causing mutations were estimated to occur in 1:217 individuals in the general Danish population.^3^

Other population studies have made similar estimates and it is generally accepted that the prevalence of FH in the general population is 1:200–250 individuals.^4–6^ In Denmark only about 12% of the individuals with FH have been identified.^4^ In other countries elaborate screening interventions have raised the rate of identified patients with FH considerably*.* ^4,7,8^

The prevalence of FH has not been directly assessed in an unselected sample of the general population in general practice clinics in Denmark. It has been shown that GPs generally have a good awareness of hyperlipidaemia and lipid-lowering therapy; however, their knowledge and awareness of national guidelines, prevalence, and diagnostic features of FH is suboptimal.^7,9^ Previous studies have shown that the prevalence of FH in general practice is underestimated.^7,10^ In addition, it is unknown whether patients with FH and hyperlipidaemia reach their treatment target when managed by GPs. From large population studies it is known that patients with FH in general are undertreated.^7,10–12^

This study aimed to provide knowledge of the prevalence and management of FH in general practice. In addition, the authors wanted to evaluate the perspective for applying an algorithm as a screening tool for FH. Furthermore, they wanted to evaluate the extent and efficacy of lipid-lowering therapy among individuals with high LDL-C levels.

## Method

This is a retrospective observational register-based study performed in collaboration with six GP clinics in the municipality of Copenhagen. The only condition for participation was use of the WinPLC system for handling electronic patient records in the clinic. This system is widely used by GPs in all of Denmark.

**Figure 1.**
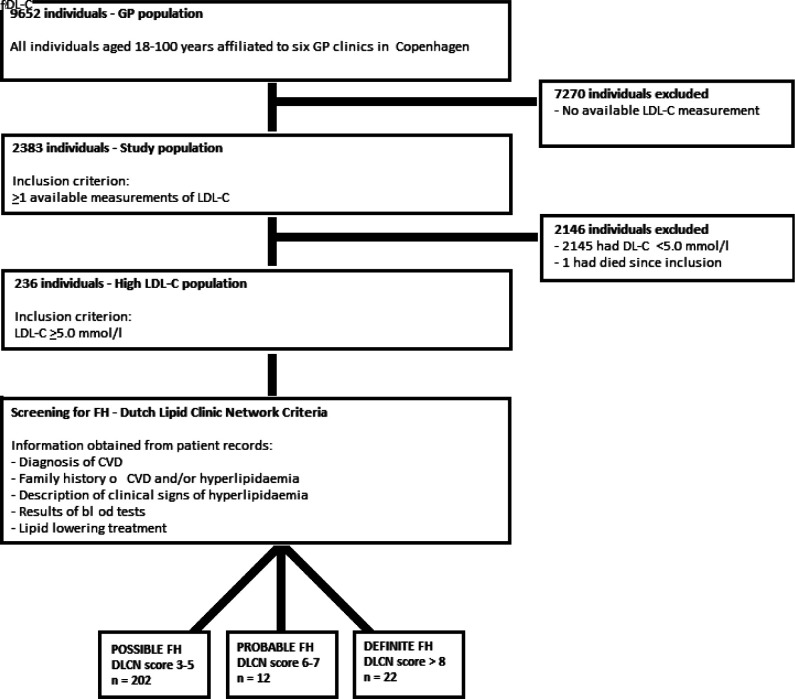
Flowchart of inclusion and exclusion throughout the study. The study population consisted of all patients with ≥1 available LDL-C measurements. The high LDL-C population (*n* = 236 individuals) consisted of patients with ≥1 LDL-C reported ≥5.0 mmol/l. CVD = cardiovascular disease. DLCN = Dutch Lipid Clinic Network. FH = familial hypercholesterolaemia. LDL-C = low-density lipoprotein cholesterol

The study population consisted of all individuals aged 18–100 years with at least one measurement of LDL-C. These were identified through the search function incorporated in the electronic patient record system (Figure 1).

Information about pre-existing CVD, diabetes mellitus (DM), hypertension, liver disease, and thyroid disease, as well as obesity and high alcohol consumption, was registered from the International Classification of Primary Care (ICPC) codes for classification of diagnoses in primary care.^13^ Information regarding lipid-lowering therapy in the study population was registered from active prescriptions in the Danish medications system.

All individuals with at least one measurement of LDL-C ≥5.0 mmol/l were then identified and included in the high LDL-C population. Individuals on lipid-lowering therapy had their latest recorded LDL-C value, regardless of level, adjusted by multiplying a correction factor according to their active treatment.^14,15^ If this pre-treatment LDL-C was ≥5.0 mmol/l the individual was included in the high LDL-C population (Figure 1). All individuals in the high LDL-C population had their patient records investigated for further information on clinical features, cardiovascular morbidity, and family medical history.

**Figure 2.**
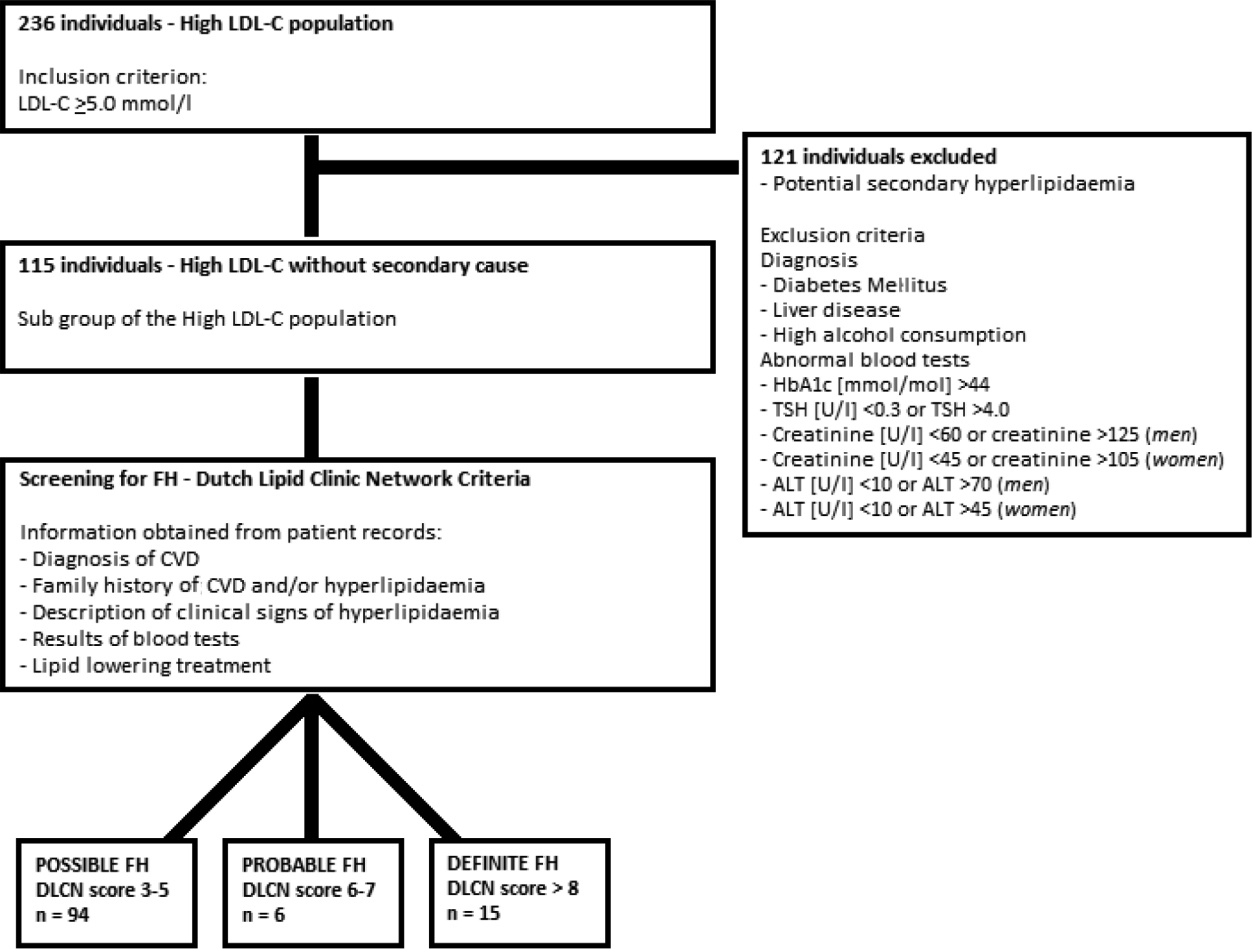
Flowchart of the analysis of the subgroup of individuals deemed to have primary hypercholesterolaemia. The high LDL-C population is defined as individuals with a reported LDL-C ≥5.0 mmol/l (Figure 1). ALT = alanine aminotransferase. CVD = cardiovascular disease. DLCN = Dutch Lipid Clinic Network. FH = familial hypercholesterolaemia. LDL-C = Low-density lipoprotein cholesterol. TSH = thyroid stimulating hormone

The high LDL-C population was assessed for potential causes of secondary hyperlipidaemia, including a diagnosis of either DM, thyroid disease, liver disease, or alcohol abuse. In addition, blood tests for glycaemic levels (HbA1c), thyroid function (thyroid stimulating hormone [TSH]), kidney function (creatinine), and liver function (alanine aminotransferase [ALT]) were evaluated. Individuals with normal values on these parameters were deemed to have potential primary hyperlipidaemia (Figure 2).

**Figure 3.**
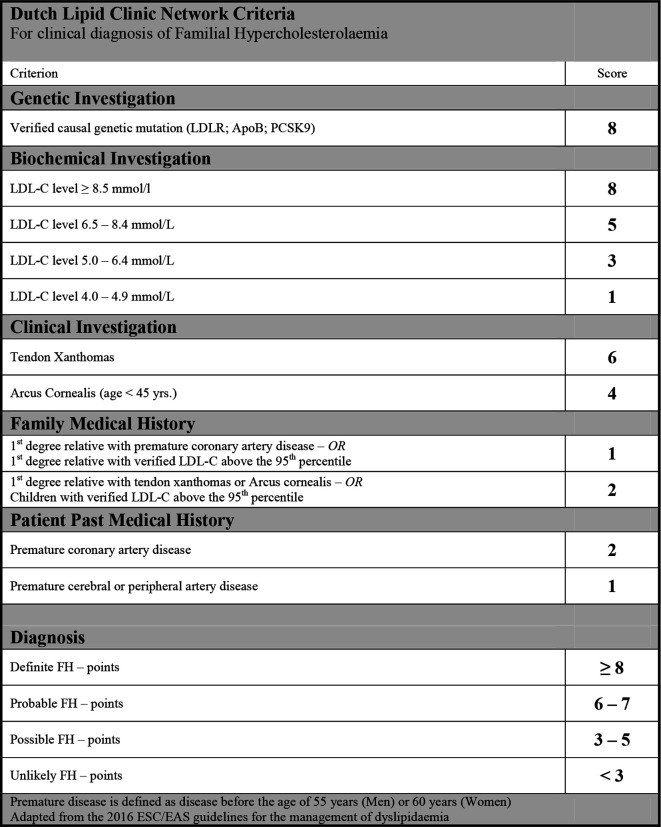
Diagnostic criteria. ApoB = apolipoprotein B. FH = familial hypercholesterolaemia. LDLR = low-density lipoprotein receptor. PCSK9 = proprotein convertase subtilisin/kexin type 9

The DLCN criteria for clinical FH of dyslipidaemia for screening was used (Figure 3).^16^

Information concerning clinical findings according to the DLCN criteria were registered as ‘met’ or ‘not met’. The highest measured value of LDL-C was used with adjustment for lipid-lowering therapy. In terms of relatives' cholesterol levels, only first-degree relatives with an estimated LDL-C above 95th percentile contributes to the patient's DLCN score, but information regarding family medical history in the patient records has been interpreted in the broadest possible way.

Tendinous xanthomata and arcus cornealis were included in the DLCN score if it was described in the medical record. Follow-up clinical confirmation was not performed as this is not allowed according to Danish legislation.

For identification of individuals with probable or definite FH, the DLCN criteria (Figure 3) was then applied to the individuals in the high LDL-C population and to the subgroup of individuals without secondary hypercholesterolaemia (Figure 2).

Furthermore, the extent and efficacy of lipid-lowering therapy were evaluated. The treatment targets were used from the 2016 European Society of Cardiology (ESC) and European Atherosclerosis Society (EAS) guidelines for management of dyslipidaemia.^16^ The number of patients reaching the actual target values were evaluated (LDL-C <1.8 mmol/l for very high risk and LDL-C <2.6 mmol/l for high risk) and the number of patients reaching a reduction of LDL-C of >50% from baseline was estimated.

### Statistical analyses

Descriptive data are presented as means with standard deviation (SD) or interquartile range (IQR). Comparison of means are calculated using *t*-tests. A *P*-value <0.05 is considered significant. All statistical analyses were performed using Stata (version 13) software.

## Results

The six GP clinics saw a total 9652 individuals between 18 years and 100 years of age (Figure 1). Of those, 2382 individuals had ≥1 measurements of LDL-C and were included in the study population. In total, 237 (9.9%) individuals had at least one measure of LDL-C ≥5.0 mmol/l. Baseline characteristics for both groups are presented in Table 1. One individual with LDL-C ≥5.0 mmol/l was deceased leaving 236 to be included in the high LDL-C population.

**Table 1.** Baseline characteristics

	**Study population, *n* (%)**	**Hypercholesterolaemia** (LDL-C ≥5.0 mmol/l), *n* %
Total	2382 (100.0)	237 (9.9)
Age, years, mean (IQR)	52.6 (19.1–98.9)	63.9 (26.9–93.4)
Sex, Male	1047 (44.0)	111 (46.8)
**Cardiovascular disease**		
Ischaemic heart disease	116 (4.9)	31 (13.1)
MI	41 (35.3)	9 (29.0)
AP	56 (48.3)	18 (58.1)
CHF	30 (25.9)	5 (16.1)
PCI	11 (9.5)	2 (6.5)
CABG	9 (7.8)	1 (3.2)
AP (no IHD)	49 (2.1)	8 (3.4)
Cerebrovascular disease	103 (4.3)	26 (11.0)
Stroke	70 (68.0)	19 (73.1)
TCI	47 (45.6)	10 (38.5)
Peripheral artery disease	49 (2.1)	10 (4.2)
**Comorbidity**		
Hypertension	454 (19.1)	115 (48.5)
Diabetes	146 (6.1)	30 (12.7)
Type 1	11 (7.5)	2 (6.7)
Type 2	135 (92.5)	28 (93.3)
Hyperthyroid disease	60 (2.5)	9 (3.8)
Hypothyroid disease	116 (4.9)	22 (9.3)
Liver disease	60 (2.5)	20 (8.4)
Viral hepatitis	18 (0.8)	2 (0.8)
Alcohol abuse	100 (4.2)	18 (7.6)
Obesity	129 (5.4)	14 (5.9)
**Medications**		
Statin	293 (12.3)	119 (50.2)
Ezetimibe	8 (0.3)	4 (1.7)
PCSK9 inhibitor	1 (0.04)	1 (0.4)
ACE inhibitor	205 (8.6)	33 (13.9)
ATII antagonist	125 (5.2)	21 (8.9)
Beta blockers	217 (9.1)	37 (15.6)
Calcium channel blockers	270 (11.3)	49 (20.7)
Diuretics	244 (10.2)	54 (22.8)

Baseline characteristics of the study population and those individuals with LDL-C ≥5.0 mmol/l. All data obtained exclusively through electronic screening of the patient records. ACE = angiotensin converting enzyme. AP = angina pectoris. ATII = angiotensin II receptor. CABG = coronary artery by-pass grafting. CHF = congestive heart failure. IHD = ischaemic heart disease. MI = myocardial infarction. PCI = percutaneous intervention. PCSK9 = proprotein convertase subtilisin/kexin type 9. TCI = transitory cerebral ischaemia.

### FH diagnostic

The DLCN criteria were applied to the 236 individuals in the high LDL-C population. Of this subgroup, 34 (14.4%) individuals were identified with probable or definite FH corresponding to 1.4% or approximately 1:70 individuals of the study population of 2382 individuals (Table 2).

**Table 2.** Identification of potential FH

	High LDL-C population (LDL-C ≥5.0 mmol/l), *n* %	High LDL-C without potential secondary cause (subgroup), *n* %
**Cardiovascular profile**		
Total	236 (100)	115 (100)
Ischaemic heart disease	41 (17.4)	24 (20.9)
MI	11 (26.8)	8 (33.3)
AP	22 (53.7)	13 (54.2)
CHF	8 (19.5)	5 (20.8)
PCI	12 (29.2)	9 (37.5)
CABG	4 (9.8)	3 (12.5)
Cerebral vascular disease	31 (13.1)	13 (11.3)
Peripheral artery disease	15 (6.4)	7 (6.1)
**Criteria for clinical FH (DLCN**)		
**Past medical history**		
Premature ischaemic heart disease	15 (6.4)	8 (7.0)
Premature cerebrovascular disease	7 (3.0)	2 (1.7)
Premature peripheral artery disease	4 (1.7)	2 (1.7)
**Clinical features**		
Tendon xanthomas	0 (0.0)	0
Arcus cornealis	1 (0.4)	1 (0.9)
**Family medical history**		
Family history of ischaemic heart disease	51 (21.6)	36 (31.3)
Family history of cerebral vascular disease	13 (5.5)	9 (7.8)
Family history of peripheral artery disease	4 (1.7)	2 (1.7)
Family history of hyperlipidaemia	27 (11.4)	17 (14.8)
No family history available	84 (35.6)	48 (41.7)
**Result of screening**		
Definite FH – DLCN score >8	22 (9.3)	15 (13.0)
Probable FH – DLCN score 6–7	12 (5.1)	6 (5.2)
Possible FH – DLCN score 3–5	202 (85.6)	94 (81.7)
**Actual diagnostic yield**		
Confirmed diagnosis of FH	3 (1.3)	1 (0.9)
FH suspected by GP	26 (11.0)	22 (19.1)

Subgroup has been derived from the high LDL-C population. Information has been obtained by reading the patient records for all the patients. AP = angina pectoris. CABG = coronary artery by-pass grafting. CHF = congestive heart failure. DLCN = Dutch Lipid Clinic Network. FH = familial hypercholesterolaemia. MI = myocardial infarction. PCI = percutaneous intervention.

After excluding all individuals with possible secondary dyslipidaemia from the high LDL-C population, a subgroup of 115 individuals were deemed to have primary hypercholesterolaemia. By applying these criteria 13 individuals with probable or definite FH were also excluded. Of the subgroup, 21 individuals (0.9% of the study population) had probable or definite FH, corresponding to 1:114 individuals.

Only 152 individuals (64.4%) in the high LDL-C population had records of a family history. Clinical signs of dyslipidaemia were described in one individual.

A total of 82 individuals (34.7%) of the high LDL-C population had been referred to treatment by a cardiologist and four of these individuals were referred to a lipid clinic. Sixty-two (26.3%) had been referred to another internal medicine outpatient clinic. The remaining 92 individuals (39.0%) had not been referred to any specialist (Table 3).

**Table 3.** Management of patients with potential FH, *N* = 236

	Result of screening, *n* %
	PossibleDLCN score: 3–5	ProbableDLCN score: 6–7	DefiniteDLCN score: ≥8
Total	202 (85.6)	12 (5.1)	22 (9.3)
**Outpatient referrals**			
Lipid clinic	0 (0.0)	0 (0.0)	4 (18.2)
Cardiologic outpatient clinic	49 (24.3)	6 (50.0)	4 (18.2)
Internal medicine outpatient clinic	57 (28.2)	1 (8.3)	4 (18.2)
Private practice cardiologist	16 (7.9)	1 (8.3)	2 (9.1)
No referral	80 (39.6)	4 (33.3)	8 (36.4)
**Lipid-lowering therapy**			
Ongoing lipid-lowering therapy	91 (45.0)	11 (91.7)	20 (90.9)
Statin therapy	88 (96.7)	11 (100)	19 (95.0)
Maximum statin dosage	49 (55.7)	5 (45.5)	10 (52.6)
Ezetimibe therapy	3 (3.4)	0 (0.0)	1 (5.3)
PCSK9 inhibitor therapy	0 (0.0)	0 (0.0)	1 (5.3)
No lipid-lowering therapy	111 (55.0)	1 (8.3)	2 (9.1)
**Diagnostic status of FH**			
FH diagnosis is made	2 (1.0)	1 (8.3)	0 (0.0)
GP suspects FH	14 (6.9)	1 (8.3)	11 (50.0)
GP does not suspect FH	186 (92.1)	10 (83.3)	11 (50.0)
**LDL-C mean mmol/l (IQR**)			
Maximum LDL-C measurement	5.20 (5.0–5.6)	5.13 (4.5–6.0)	5.84 (5.3–6.1)
Pre-treatment LDL-C	5.63 (5.2–5.9)	6.82 (6.4–7.3)	9.75 (8.8–10.7)
Latest LDL-C measurement	3.73 (2.8–4.8)	3.44 (3.1–4.1)	4.77 (4.1–5.5)
No lipid-lowering therapy	4.4 (3.8–5.2)	3.6 ()	3.3 (3.1–3.5)
Lipid-lowering therapy, overall	2.9 (2.5–3.4)	3.4 (3.0–4.3)	4.9 (4.6–5.6)
Statins	2.9 (2.5–3.4)	3.4 (3.0–4.3)	4.8 (4.4–5.5)

All patients are from the high LDL-C population. DLCN = Dutch Lipid Clinic Network. FH = familial hypercholesterolaemia. LDL-C = low-density lipoprotein cholesterol. PCSK9 = proprotein convertase subtilisin/kexin type 9.

### Medical treatment

Lipid-lowering therapy, predominantly statins, were prescribed to 122 individuals (51.7%) in the high LDL-C population. The proportion of prescribed lipid-lowering therapy tended to be higher in the groups with probable and definite FH compared with the entire high LDL-C population. Furthermore, 33 individuals had previously been on lipid-lowering therapy, but for some reason they had come off their treatment (data not shown). Ezetimibe was prescribed to four individuals and only one individual was prescribed PCSK9 inhibitors (Table 3). Of 118 individuals in the high LDL-C population receiving statins, 64 (54.4%) individuals were receiving maximum dosage of statins.

Among patients with CVD (*n* = 68) in the high LDL-C population, 53 (77.9%) received lipid-lowering therapy but only three (4.4%) reached the treatment target of LDL-C <1.8 mmol/l, according to the 2016 ESC and EAS guidelines for the management of dyslipidaemia.^16^ It was found that 54.9% of patients with CVD who were treated with statins were receiving maximum dosage. Among individuals with high LDL-C and no CVD (*n* = 168) the corresponding values were 41.1% (*n* = 69) receiving lipid-lowering therapy and 13.1% (*n* = 22) reached the target of LDL-C <2.6 mmol/l (Table 4). Among patients with CVD and high LDL-C 37 (53.9%) reached reduction of LDL-C of >50%, and among patients without CVD 39 (23.4%) reached a 50% reduction (data not shown).

**Table 4.** Evaluation of lipid-lowering therapy

	High LDL-C population (LDL-C ≥5.0 mmol/l),*n* = 236, *n* %	Hypercholesterolaemia without potential secondary cause (subgroup),*n* = 115, *n* %
**Patients with CVD diagnoses**		
**Treatment target** **—** **LDL-C** **>** **1.8** **mmol/** **l**		
Total number of patients	68 (28.8)	34 (29.6)
Patients receiving statins	51 (75.0)	26 (76.5)
Other lipid-lowering therapy	2 (2.9)	0 (0.0)
Maximum dosage of statins	28 (54.9)	15 (57.7)
Patients reaching treatment target^a^	3 (4.4)	2 (5.9)
**LDL-C mean mmol/l (IQR**)		
Maximum registered	5.16 (4.90–5.60)	4.94 (5.00–5.40)
Adjusted pre-treatment	6.36 (5.36–6.73)	6.51 (5.30–7.18)
Latest follow-up	3.31 (2.50–4.10)	3.44 (2.60–4.20)
**Patients without CVD diagnoses**		
**Treatment target – LDL-C** **>** **2.6** **mmol/** **l**		
Total number of patients	168 (71.2)	81 (70.4)
Patients receiving statins	67 (39.9)	22 (27.2)
Other lipid-lowering therapy	2 (1.2)	0 (0.0)
**Maximum dosage of statins**		
Overall	36 (53.7)	15 (68.2)
Possible FH	28 (77.8)	10 (66.7)
Probable FH	3 (8.3)	1 (6.7)
Definite FH	5 (13.9)	4 (26.7)
**Patients reaching treatment target**		
Overall	22 (13.1)	6 (7.4)
Possible FH	22 (100)	6 (100)
Probable FH	0 (0.0)	0 (0.0)
Definite FH	0 (0.0)	0 (0.0)

Subgroup has been derived from the high LDL-C population. ^a^Treatment target as defined in the 2016 European Society of Cardiology (ESC) and European Atherosclerosis Society (EAS) guidelines for management of dyslipidaemia.^16^ CVD = cardiovascular disease. FH = familial hypercholesteroleamia. LDL-C = low-density lipoprotein cholesterol.

## Discussion

### Summary

This study examined the prevalence and management of FH in general practice. The main findings included that there is still a massive lack of recognition of FH in general practice. Despite a high LDL-C being measured, the diagnosis was rarely made, not even if the patient had a cardiovascular event. Of the patients undergoing treatment, only a few of them reached their treatment targets, according to ESC and EAS guidelines for the management of dyslipidaemia. Only about half of the patients received statins, many in low doses, and only a few were treated with ezetimibe. Only one patient was treated with PCSK9 inhibitor.

Overall, 34 individuals were identifed with probable or definite FH, according to the DLCN criteria. With a general estimate of an incidence of 1:200–250, the six clinics should have between 38 and 48 patients with FH. However, individuals were excluded with potential secondary hypercholesterolaemia, which dismissed 13 individuals with probable or definite FH (38.2%).

In this study the FH was diagnosed using the DLCN criteria as these criteria are recommended in order to establish the clinical diagnosis of FH.^10,17^

The study found a low number of patients who reached their treatment target. If a reduction of LDL-C of >50% is applied as a criterion for reached treatment target, then 37 (53.9%) of the patients with CVD reached their treatment target. Among the patients with high LDL-C but no CVD, 39 patients (23.4%) reached their treatment target. This figure, however, must be interpreted with caution, as many patients receiving statins have no pre-treatment LDL-C available. In these cases, the pre-treatment LDL-C is estimated from the effect of their lipid-lowering therapy, and the reduction, therefore, reflects this estimation.

### Strengths and limitations

This study relies solely on data provided by records at the GP clinics. The familial history or the past medical history could not be further elaborated on than the available information in patient records, and there was no possibility to verify the LDL-C measurements by follow-up blood tests.

According to the DLCN criteria, FH should be suspected already at a low-density lipoprotein (LDL) level at 4.0 mmol /l. The threshold of 5.0 mmol/l was chosen, since a considerable proportion of the individuals with an LDL-C between 4.0 and 4.9 mmol/l will have secondary hypercholesterolaemia. This, of course, will exclude patients with FH with LDL-C levels between 4.0 and 4.9 mmol/l, thereby causing an underestimation of the number of potential patients with FH.

The extent of the true missing values in this dataset cannot be evaluated precisely. The records only reflect positive findings. There might also be an underestimation of registering positive findings, such as tendon xanthomas, arcus cornealis, and family history, thereby underestimating the number of patients with FH.

Only two-thirds of the high LDL-C population had familial medical history available, which will lead to underestimating the prevalence of FH in this setting. However, this proportion of registration is relatively high compared with other studies.^18–20^

Information regarding first-degree relatives and their diagnoses was obtained from the patient records when mentioned. According to Danish legislation, the records of the patient’s relatives cannot be investigated. Therefore, information on CVD or hypercholesterolaemia was entered in the study as a fulfilled criterion of premature CVD or hypercholesterolaemia, even if the age of the relative was not noted. This will of course overestimate the DLCN diagnostic score, but the approach was chosen in order to identify as many individuals with potential FH as possible.

There is a considerable lack of registration, especially of CVD diagnoses, which is a critical component in the clinical diagnosis of FH. The study found 2.9% in the entire population (*n* = 9652) versus approximately 9% as generally estimated in the Danish population.^21,22^

The study population concerns only a quarter (*n* = 2382, 24.6%) of the entire 9652 individuals and there is no information on the remaining part of the population. However, one must expect that those included in the study population are individuals with symptoms or suspicion of possible sickness; therefore, the incidence of potential FH may be lower in the rest of the group, but, to the authors’ knowledge, there are no data to support this thesis.

LDL-C is not measured routinely in general practice in Denmark. The measurement is only performed if the clinical examination of an individual or an individual’s risk factors warrants this. The individuals in this study, therefore, might not be a fully random sample of the population, as they have been selected for lipid measurement by their GP owing to medical history or clinical condition.

### Comparison with existing literature

It was found that only one-third of the high LDL-C population had been referred to any cardiology specialist and only four individuals (1.7%) to a lipid clinic. These four patients were all deemed to have definite FH. Subsequently, nearly two-thirds of individuals with probable or definite FH (61.8% with DLCN score >5) were neither diagnosed with, nor suspected of having, FH. This vast majority of individuals with FH were not being identified in primary care, this has also been found in other studie*s.* ^23,24^

In this study screening within the primary healthcare database was successful in detecting a provisional diagnosis of FH. This is in line with what has been found in other studies.^25–28^

Numerous definitions have been suggested during the last decade on adequate treatment in FH, but no evidence-based clinical trials on treatment goals exist. At the time of data collection in this study, the recommendation in Denmark was the same as in the ESC 2016 guidelines on CVD prevention in clinical practice: high risk <2.6 mmol /l (<100 mg/dl) or a reduction of at least 50% if the baseline is between 2.6 and 5.2 mmol/l (100–200 mg/dl); and for very high risk <1.8 mmol/l or reduction of at least 50% if the baseline is between 1.8 and 3.5 mmol/l (70–135 mg/dl).^16,29^

This lack of sufficient treatment is illustrated by the levels of LDL-C, which remained high even in the high-risk individuals. The proportion of patients reaching absolute LDL-C targets was low as well: among patients with CVD only 4.4% reached the treatment target of LDL-C <1.8 mmol/l, even though 77.9% received lipid-lowering therapy. In this population 53.9% of the patients with CVD had their baseline LDL-C value reduced by >50%. This means that almost half of the patients did not reach their treatment target.

Despite the fact that many individuals do not reach their treatment target, only half of the high LDL-C population were on lipid-lowering therapy. These findings correspond well with findings in a previous population study.^10^ In addition, less than one-third of the individuals in the high LDL-C population receiving statins were treated to maximum dosage. This emphasises a need for better care. Only a few patients received ezetimibe but this may reflect that ezetimibe was only eligible for a subsidy from the state in 2018 and, thus, during the study period was quite expensive compared with statins. In Denmark, PCSK9 inhibitors are dispensed free of charge, but only cardiologists, endocrinologists, and neurologists can prescribe PCSK9 inhibitors. The state has set a limit for LDL of >3–4 mmol/l depending on the underlying disease and patients must be treated to the maximum tolerated dose of statin and ezetimibe before PCSK9 inhibitors can be delivered.

These factors may have influenced the level of treatment. At present, the 2019 ESC and EAS guidelines for the management of dyslipidaemia establishes a treatment target of LDL-C <1.4 mmol/l for individuals at very high risk and LDL-C <1.8 mmol/l for individuals at high risk.^17^ In this context, most of the individuals in this population would probably fail to reach their treatment target.

### Implications for research and practice

In this study the possibility of determining the prevalence of FH from pre-recorded information has been examined. The WinPLC patient record system was used for identifying diagnoses and levels of biochemical markers before manually reading through the patient records. It has been shown that clinical case-finding algorithms, such as TARB-Ex system and FAMCAT, are useful in identifying patients in primary care suspected of FH.^23,30,31^ The study identified several potential patients with FH, which strongly suggests that a similar system could be a valuable addition to the Danish electronic patient records in general practice. The use of electronic reminders combined with potential patients with FH being identified by electronic data extraction at the general practice has shown to raise the rate of referred patients for further diagnostics at a specialist lipid clinic.^32^

In collaboration with the biochemical laboratories, a high LDL could automatically be accompanied by a marker stating that individuals with a high LDL should be suspected of FH and family investigation considered.^27,33^ Similarly, an electronic message could automatically be forwarded to the general practice by the electronic patient record system whenever a diagnosis of, for instance, MI, ischaemic heart disease, or stroke is registered to an individual aged <55–60 years in order to identify potential FH and initiate cascade screening. A combination of a registered high LDL-C and a diagnosis of CVD at a young age could also trigger an automatic notification. Such electronic aids could serve to raise awareness of potential FH among GPs.

Over the last decade, there has been considerable focus on FH and many initiatives have already been launched. A global overview of the current status of FH has been published recently and guidelines for detecting and treating FH have been provided.^4,10^ As a result of this and other initiatives, the number of diagnosed patients with FH must be expected to increase. In Denmark the estimated number of diagnosed patients with FH has increased from about 4% to at least 13% in a few years.^4,10^ However, this study confirms that there is still a massive lack of recognition and treatment of FH in general practice. Primary care has a key role in diagnosis and significant potential exists in primary care to identify new cases of FH who could also act as new index cases for a family screening programme. Studies have shown that GPs consider themselves to be a key figure in diagnosing FH, but there is a lack of knowledge on how to handle these patients.^34,35^

There is a need for an education programme and a better diagnostic work-up including collaboration with the biochemical laboratories. Thus, several screening options have been suggested, including a clinical case-finding algorithm, a marker when a high level of LDL-C is registered on a blood test, or when diagnosis of CVD at a young age is registered. Support from other providers within the primary care setting or triaging to genetics healthcare professionals is needed.^2^ Finally, there is a need to increase awareness of FH in the general population so that the population becomes aware of the importance of a health check if there are family members with high cholesterol or early CVD. Future research is desired to evaluate a broader implementation of the above regarding detection of FH in general practice, early treatment, and the effect on cardiovascular outcomes.
